# Improving the quality and quantity of clinical and translational research statewide: An application of group concept mapping

**DOI:** 10.1017/cts.2020.572

**Published:** 2021-01-05

**Authors:** Jacquelyn Fede, Stephen J. Kogut, Anthony Hayward, John F. Stevenson, Cynthia Willey-Temkin, Heather Fournier, Gabrielle Stranieri, Judy A. Kimberly, James Padbury

**Affiliations:** 1 The University of Rhode Island, College of Health Sciences, Kingston, RI, USA; 2 Advance-CTR, Providence, RI, USA; 3 The University of Rhode Island, College of Pharmacy, Kingston, RI, USA; 4 Brown University, Division of Biology and Medicine, Providence, RI, USA

**Keywords:** Clinical translational research, evaluation, mixed methods, stakeholder engagement, qualitative methods

## Abstract

**Introduction:**

Advance Clinical and Translational Research (Advance-CTR) serves as a central hub to support and educate clinical and translational researchers in Rhode Island. Understanding barriers to clinical research in the state is the key to setting project aims and priorities.

**Methods:**

We implemented a Group Concept Mapping exercise to characterize the views of researchers and administrators regarding how to increase the quality and quantity of clinical and translational research in their settings. Participants generated ideas in response to this prompt and rated each unique idea in terms of how important it was and feasible it seemed to them.

**Results:**

Participants generated 78 unique ideas, from which 9 key themes emerged (e.g., *Building connections between researchers*). Items rated highest in perceived importance and feasibility included providing seed grants for pilot projects, connecting researchers with common interests and networking opportunities. Implications of results are discussed.

**Conclusions:**

The Group Concept Mapping exercise enabled our project leadership to better understand stakeholder-perceived priorities and to act on ideas and aims most relevant to researchers in the state. This method is well suited to translational research enterprises beyond Rhode Island when a participatory evaluation stance is desired.

## Introduction

Experts in medical and health policy recognized in the early 2000’s that despite advances in basic biomedical research, the movement of laboratory innovations to patient-relevant treatments and public health arenas was lacking. This realization stimulated a variety of efforts, particularly inside the National Institutes of Health (NIH), to identify and overcome barriers to the transfer of scientific knowledge in the health realm [1]. One significant initiative continues to direct funding to states with relatively low levels of current NIH support, as part of the Institutional Development Award (IDeA) process [2]. To enhance competitiveness for clinical and translational research (CTR), grants have been awarded to build infrastructure and human resources in the form of pilot project funding, support for biostatistical and clinical research services and for providing training and mentorship opportunities. The Advance-CTR program provides awards, services and training to Rhode Island-based medical and health researchers. The program began in 2016 with support from an IDeA-CTR award through the National Institute of General Medical Sciences (NIGMS) [3], with a scope that includes the state’s two major research universities, Brown University and the University of Rhode Island, together with the Rhode Island Quality Institute, which houses Rhode Island’s state healthcare information exchange and the three major hospital systems in Rhode Island, Lifespan, Care New England, and the Providence Veterans Affairs (VA) Medical Center.

Evaluators have been called upon to inform these NIH initiatives and to develop methods that are responsive to the context while bringing in standards and principles from the field of evaluation science [4]. A designated team of internal evaluators is responsible for tracking progress and evaluating the program’s effectiveness. A CTR program’s resources must be aligned to the needs of researchers, research administrators, and a range of institutional contexts to best address the foremost challenges of the translation of clinical research from discovery to utilization throughout the state. Program theory for this project must involve multiple organizations, often across considerable distances, and with varying organizational and professional cultures, bureaucratic processes and hurdles, and staff priorities. It also must be able to account for complex and dynamic chains of causality which are difficult to evaluate rigorously [5]. Finding effective strategies that can work to overcome the challenges to stakeholder engagement and make evaluation valid, responsive, and influential for examining outcomes and for continuing to plan the program is challenging and explicit processes are not agreed upon in the field [6–8].

From the outset, we have taken a participatory approach, employing various strategies to engage with the full range of program stakeholder groups as we developed and conducted our evaluation plan [9]. This paper examines one important strategy we have used to engage our community of researchers and clinicians as active participants and informants of their perceived challenges, barriers and needs for support.

### Group Concept Mapping

Group Concept Mapping (GCM) is a validated approach to inform decision making and is especially useful in contexts involving diverse stakeholders and complex issues. It is a mixed methods approach that combines qualitative idea generation to an open-ended focus prompt with quantitative clustering and rating of generated ideas. In this way, it is a stakeholder-authored approach that corroborates input across varied perspectives and stakeholder priorities. Further, the process allows for using highly intuitive visualizations of results that are easily comprehended and more likely to be used [10,11]. GCM has been applied within Clinical and Translational Science contexts; for example, to map a research agenda for evaluating team science [12], and to conceptualize trust in community-academic research partnerships [13]. Earlier work by Robinson and Trochim [14] employed this approach to examine barriers to participation of minority populations in clinical trials studies of medical interventions. Additionally, other efforts have suggested that for diverse and complex multi-organizational contexts, GCM works well to guide logic model development [15].

We used GCM as a follow-up to a prior online needs assessment survey to gain a better understanding of how Advance-CTR can accelerate clinical and translational research in Rhode Island, particularly during the initial years of the program [16]. This paper describes our application of the GCM methodology to inform the leadership of our CTR and our partner institutions about priorities and opportunities for enhancing clinical and translational research in our state, as perceived by the communities of researchers, clinicians, and institutional administrators served by Advance-CTR. The findings have also informed our evolving logic model for this program and planning and prioritizing of future initiatives. Investigation of the generalizability of these specific results to other CTR and CTSA settings will be a productive extension of this work.

The illustrative results reported here include a description of the ideas and themes rated by stakeholders according to their importance and feasibility and comparisons of these ratings across key subgroups. We also discuss how our results were disseminated and applied and share lessons learned regarding the advantages and limitations of GCM as an evaluation component for an IDeA-CTR program and for other similarly large-scale and complex organizational contexts.

## Methodology

### Participants

There is no minimum threshold of statements required for Group Concept Mapping; however, a minimum of 40 participants at each stage of the process and at least 10 participants per cell for any between group comparisons is needed [10]. In order to recruit for this project, clinical and translational researchers, together with relevant staff and administration from all the affiliated institutions of the project, were identified by program administrative staff and contacted by email with an invitation to participate. The email solicitation was sent to approximately 3000 recipients through email listservs. Of those, 119 participated in online brainstorming and 57 participated in in-person sorting and rating at a statewide retreat.

Table 1 presents the sex, affiliation, position/role, and research experience of participants in the online idea generation and the on-site sorting and rating tasks. The two samples illustrate the range of participants and show that the two groups were sufficiently diverse to represent our stakeholders adequately. There were more female than male respondents among idea generators (57.1% versus 25.2%, respectively), and sorter/ raters (50.9% versus 29.8%, respectively), while Assistant Professor was the modal rank of idea generators (23.5%) and sorter/raters (26.3%). Hospital-based staff were less represented at the sorting-rating task (19.3% of participants). Of note, 41.2% of idea generators and 36.8% of sorter/raters had no previous federal funding as primary investigator.

**Table 1.** Demographics of participants in idea generation and sorting and rating tasks

	Idea Generation		Sorting and Rating	
	*N*	%	*N*	%
**Sex**				
Female	68	57.1	29	50.9
Male	30	25.2	17	29.8
Not recorded	21	17.6	11	19.3
**Affiliation**				
Brown University	26	21.8	14	24.6
University of Rhode Island	37	31.1	20	35.1
Hospital-based	33	27.7	11	19.3
Other/not recorded	23	19.3	12	21.1
**Rank/Position**				
Assistant Professor	28	23.5	15	26.3
Associate Professor	14	11.8	7	12.3
Full Professor	25	21.0	11	19.3
Administrator	9	7.6	6	10.5
Staff	14	11.8	3*	5.3
other/not recorded	29	24.4	15	26.3
**Research Experience**				
No previous federal funding as primary investigator	49	41.2	21	36.8
Total funding up to $150,000 as primary investigator	14	11.8	7	12.3
Total funding exceeds $150,000 as a primary investigator	40	33.6	19	33.3
Did not respond	16	13.4	10	17.5

* Staff were included with Administrators in analyses due to small cell size and similarity in roles.

### Materials and Methods

The Group Concept Mapping process begins with the identification of a “focus prompt,” that is, a key question to which participants respond, generating the ideas that then drive all subsequent steps of the process. Ideas generated in response to the focus prompt are reviewed and synthesized, and then presented for sorting and rating, followed by analysis and presentation of results [17]. These steps were facilitated using the CS Global Max^TM^ [18] online tools developed by Concept Systems Incorporated. The idea brainstorming process was performed online during the two months that preceded an in-person retreat during which the sorting and rating tasks were completed using the generated ideas. Sorters and raters included both individuals who had participated in brainstorming as well as other retreat attendees who had not. Analyses and presentation of results occurred in the months following the retreat, with an emphasis on getting the results considered and used by stakeholders having the power to affect change, as described in the discussion section of this report.

Our focus prompt: “*To increase the quality and quantity of clinical and translational research in Rhode Island, we should…”* was developed by the Tracking and Evaluation team in consultation with experienced clinical researchers from the Advance-CTR program, and with guidance from consultants at Concept Systems Incorporated. This prompt aligned closely with the goals of the Advance-CTR program, which aims to extend resources for enhancing translational research according to the needs of researchers in Rhode Island. Researchers’ needs were previously elucidated during the project’s participatory kick-off retreat and from a needs assessment survey conducted during the prior year.

In Group Concept Mapping, the study outcome is determined by the populations selected to participate. Therefore, we aimed to distribute our focus prompt by email as widely as possible across our participating institutions. We anticipated that respondents would self-select as stakeholders in translational science, and we specifically included all levels of research administrators as a key stakeholder group responsible for decisions ranging from resource allocation to policies, communications and support delivery.

Upon opening the link, participants were presented with a series of demographic questions (i.e., institutional affiliation, academic rank or role, prior research funding experience, sex, and role within or use of Advance-CTR services and programs). The demographic indicators were selected to represent factors that could theoretically influence perception of barriers to research. Once demographic questions were submitted, respondents then anonymously shared their ideas for how the quality and quantity of CTR could be improved in their setting. Participants were encouraged to submit as many ideas as they could think of, and no boundaries were provided regarding the range, specificity, domain, length, or generalizability of an idea to other researchers. The brainstorming period took place from the middle of October through early November of 2017, with participation encouraged through the Advance-CTR newsletter and with periodic email reminders to submit their feedback.

The ideas generated during brainstorming were then reviewed by the project team in preparation for the sorting and rating activities. Duplicate themes were consolidated into single items, ideas that were considered too specialized or individualized for broad consideration were deleted, and complex statements were broken into single ideas (e.g., the statement “we should have more networking events and get more protected time for research” would become two separate ideas). At a retreat in December of 2017, participants working individually on laptops participated in GCM’s sorting and rating activities. During the sorting task, participants grouped all unique ideas into clusters as they saw fit according to similarity in meaning alone. This process was carried out using the software program, which allows the generated ideas to be dragged and dropped into thematic clusters. At least two clusters were to be formed (i.e., one-cluster models are not allowed), but there was not an upper limit to how many clusters could be created. In addition, participants were required to name each of their created clusters based upon their conceptualization of how the clustered ideas were related. In a separate activity, retreat participants were then asked to rate each idea according to its perceived importance, and then according to its perceived feasibility, using a 5-point Likert scale ranging at lowest from “not at all important/feasible” to highest as “extremely important/feasible.” When the rating task was completed, the retreat attendees were thanked for their participation and told that the results of the group concept mapping activity would be shared with them at a later time.

The GCM software application uses the clustered and rated ideas to create a point map through multidimensional scaling [10,17], which illustrates the spatial relationship of items; items that were closer in proximity on the point map had been more frequently sorted together (i.e., placed in the same cluster) by participants. The software, using hierarchical cluster analysis [10,17], then analyzed the point map to identify a range of possible cluster structure solutions, and the evaluation team balanced parsimony with interpretability to choose a cluster structure set for further analyses. The participants’ ratings of the items within these clusters were averaged to determine the overall importance and feasibility of clusters as well as the average rated importance and feasibility for each individual statement. Statements were also plotted on the dimensions of importance and feasibility to determine which statements, on average were rated as the most important and the most feasible by the research community. Follow-up analyses used the ratings to compare perceptions of different subgroups of participants. Graphic representations were produced by the software with the intention to stimulate discussion of project priorities.

This study was approved by the Brown University and University of Rhode Island Institutional Review Boards.

## Results

In response to our emailed focus prompt, we received 150 brainstormed statements from 75 respondents for ideas to enhance CTR in Rhode Island. Following the procedure described above, the 150 statements were then culled by our project team to yield 78 unique items. These ideas were then sorted and rated by the 57 self-selected participants who chose to attend a half-day retreat. Table 1 shows participant characteristics at both the brainstorming and sorting and rating stages of the project.

**Fig. 1.**
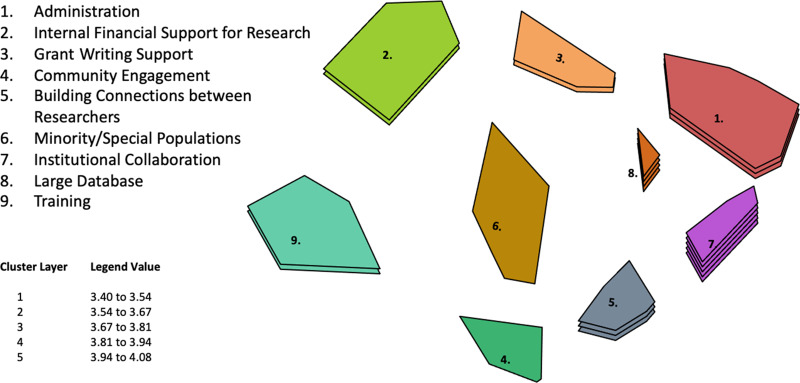
Cluster Model of Themes for Improving the Quality and Quantity of Clinical and Translational Research with Overall Importance (Clusters with more layers were rated more important).

Figure 1 presents the cluster model chosen as best representing the point map of generated ideas. This model contains 9 themes, with titles generated as: internal financial support for research; grant writing support; administration; training; minority and special populations; large databases; institutional collaboration; community engagement; and connections between researchers. Table 2 provides the statements grouped by theme to clarify the content represented by theme titles.

**Table 2.** Statements in each cluster with mean Importance (Imp.) and Feasibility (Feas.)

Cluster	Statement (ST)	*M* Imp.	*M* Feas.
**1. Administration**			
1	Invest in modernized systems for grants administration	3.96	3.60
3	Enhance facilities support	3.62	3.53
4	Improve post-award administrative support	3.80	3.84
5	Ensure that overlapping departments have Standard Operating Procedures or best practices available to all researchers	3.39	3.54
11	Streamline animal use application process	2.98	3.09
14	Improve pre-award support for grant applications	4.18	3.72
28	Have a common Institutional Review Board (IRB) and Institutional Animal Care and Use Committee (IACUC) between Brown, Lifespan hospitals, and Care New England (CNE) hospitals	4.20	3.09
41	Expedite the review and approval of applications	3.82	3.34
42	Expedite contract negotiation	3.60	3.06
44	Improve contact and communication with our grants management offices and staff	3.77	3.85
51	Streamline the regulatory review process via electronic “one-stop-shop” committee submission process	3.89	3.23
53	Create a pre-IRB consult service	3.65	3.89
67	Improve support for budgeting	3.47	3.76
73	Identify strengths and weaknesses of sponsored projects services	3.45	3.92
**2. Internal Financial Support for Research**			
2	Provide seed grants to encourage new collaborations across basic science and clinical faculty	4.39	4.36
7	Offer scholarships for publication fees so we can extend research findings to reach new audiences	3.05	3.57
13	Increase hard money support for research faculty	3.80	2.77
16	Provide more small to moderate funding opportunities that do not require full grant application	3.96	3.69
18	Increase the labor supply of fellows and postdocs to perform clinical translational research through advertised funding	3.52	2.96
20	Prioritize Small Business Innovation Research (SBIR) and Small Business Technology Transfer (STTR) awards	3.09	3.28
21	Increase funding support for established investigators	3.25	2.89
25	Provide pilot funding for multi-principal investigator (PI) projects	4.11	3.87
30	Enhance and incentivize participation of clinical faculty in research	3.75	3.39
52	Provide funds for more senior investigators to attend National Institutes of Health (NIH) and National Science Foundation (NSF) workshops	3.05	3.06
55	Offer better benefit packages to attract and retain scientists in Rhode Island (RI)	3.66	2.69
70	Provide funding for moving basic science to translational path	3.79	3.24
76	Provide faculty with release time	3.80	2.89
**3. Grant-Writing Support**			
34	Improve avenues to commercialization of research innovations	3.20	3.13
43	Augment pre-application (e.g., R01, K23, K01) counseling and review	3.96	3.58
48	Have pre-submitted grants evaluated by mock study sections	3.64	3.61
60	Preserve existing research lab facilities and resources	3.69	3.34
61	Improve support for grant writing	4.18	3.98
63	Address funding ineligibility for researchers supported by other programs	2.95	3.06
**4. Community Engagement**			
6	Hold monthly brainstorming sessions among clinical and translational research (CTR) community	2.96	4.15
17	Consult community stakeholders to identify key health needs	3.59	3.83
19	Ensure patients are able to provide insight and input into research and practice	3.43	3.43
23	Identify and promote local research strengths associated with RI health priorities	3.98	4.17
24	Involve patients and families in research	3.48	3.43
69	Have clinicians and researchers shadow each other for a day	3.14	3.22
**5. Building Connections Between Researchers**			
8	Identify clinical and translational research areas that leverage the basic science and clinical strengths across institutions	3.93	4.15
15	Have a speed dating event for scientists to foster connections and collaborations	3.48	4.25
29	Create partnerships with local clinics that serve the Rhode Island (RI) community	3.86	3.44
35	Connect researchers with common interests	4.18	4.31
36	Configure research teams based on potential to advance research	3.91	3.80
39	Create a unified statewide directory of researcher expertise and projects	4.11	3.96
47	Create recurring networking opportunities that connect researchers from different domains	4.13	4.13
54	Develop more effective ways of identifying external collaborators	3.93	3.70
64	Form subgroups for specialized themes within CTR	3.40	4.19
71	Create discussion groups between local Centers of Biomedical Research Excellence (COBRE) investigators	3.36	3.93
**6. Minority/Special Populations**			
12	Emphasize CTR in particular theme areas	3.16	3.85
27	Help researchers with childcare and eldercare needs	2.85	2.62
38	Provide “how to” assistance and point-of-contact information (roles/responsibilities) for collaboration	3.64	4.00
45	Better promote intellectual, technical and analytical resources available to the community	3.82	3.72
56	Enhance promotion of published research	3.59	4.06
72	Improve recruiting of participants for studies	3.53	3.34
**7. Institutional Collaboration**			
31	Encourage colleges/departments to share resources and materials	4.11	3.74
33	Increase collaboration between participating institutions for budget and reporting purposes	3.93	3.37
40	Put in place mechanisms that will help both Brown University and Unversity of Rhode Island collaborate without competing	3.95	3.26
59	Create blanket collaboration agreements between institutions within the RI CTR network	4.18	3.43
75	Reduce administrative barriers to collaboration	4.32	3.39
**8. Large Database**			
57	Provide access to a centralized site that securely houses large datasets	4.04	3.70
58	Improve access to analytic software	3.95	3.92
65	Make RI Health Information Exchange data available to researchers	3.96	3.72
74	Better utilize research tools and procedures developed in the private sector	3.48	3.22
**9. Training**			
9	Create research training courses for those in the CTR community	3.66	4.17
10	Provide trainings, webinars, recorded talks, and other resources in online formats	3.59	4.39
22	Train junior researchers to effectively and appropriately utilize mentor time and guidance	3.79	3.76
26	Identify successful female researchers to serve as role models	3.71	4.38
32	Train clinical researchers to analyze healthcare databases	3.74	3.46
37	Provide advanced REDCap workshops	3.27	4.42
46	Ensure that junior faculty have experienced mentors with a track record of funding	4.23	3.93
49	Provide ongoing educational opportunities for administrators	3.45	3.66
50	Provide more REDCap workshops	3.20	4.19
62	Develop a team approach to mentoring so that new researchers can benefit from both R01-experienced mentors and subject-specific mentors at the same time	4.04	3.91
66	Sponsor keynote speakers on different themes	3.02	4.29
68	Get medical/clinical students involved early so that they get “the bug” for research	3.44	3.70
77	Not underestimate how intimidating the research process is for people just starting out and how easily people can give up!	3.34	3.87
78	Offer science communication courses that focus on oral and written formats directed at lay audiences and scientists in other fields	3.59	3.96

*Note:* M imp. and M feas. represent the mean importance rating and mean feasibility rating, respectively.

**Fig. 2.**
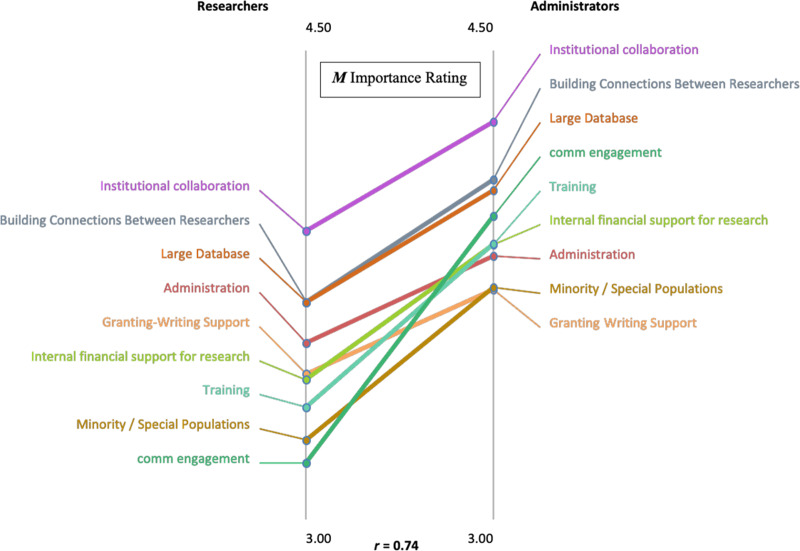
Pattern Match Diagram of Clusters Ranked by Absolute Ratings of Importance: Researchers Compared with Administrators.

A “pattern match” diagram (Fig. 2) illustrates our use of this graphic, showing differences between researchers’ and administrators’ ratings of the perceived importance of ideas within each theme. We made several similar comparisons between subgroups of the retreat participants, also useful for prompting follow-up consideration, as discussed below. Both groups ranked inter-institutional collaboration as the top priority, followed by building connections among researchers. Elucidating some differences, administrators generally rated the importance of these aids to CTR higher than researchers did. In particular, administrators ranked community engagement substantially higher than researchers did, while administration and grant writing support were seen as relatively more important by researchers than administrators. Nevertheless, cluster item rankings between researchers and administrators were strongly correlated (*r* = 0.74).

**Fig. 3.**
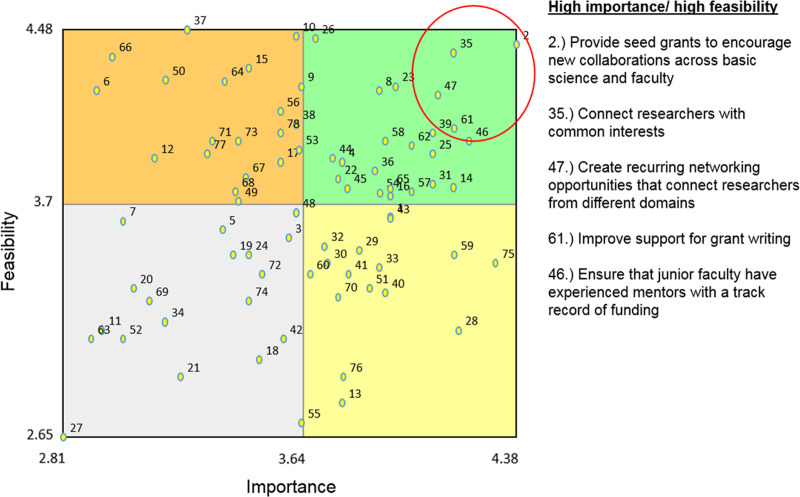
Go Zone Map: Importance and Feasibility of Generated Ideas. *Note:* Both the Importance and Feasibility scales ranged from 1 (not at all important/feasible) to 5 (extremely important/feasible).

Another useful graphic presentation of our data was “go zone” quadrant mapping (Fig. 3). This chart identifies items that were rated higher and lower according to the dimensions of importance and feasibility. Feasibility is depicted on the vertical axis and importance is depicted on the horizontal axis. The items within the upper right quadrant were those rated highest on both dimensions. Particularly important and feasible items are circled, and include providing seed grants to encourage new collaborations across basic science and clinical faculty, and connecting researchers with common interests. Grant writing support and mentoring were also highly rated. The lower right quadrant comprises items that were considered highly important but thought to be less feasible. These include reducing administrative barriers to collaboration and having a common Institutional Review Board (IRB) across institutions.

These results were also analyzed according to subgroups of interest, and we provide salient highlights here (supplementary figures available upon request). We considered a comparison across the participating organizations to be of particular interest given the concern for inter-institutional collaboration. At our public university, support for grant writing and the availability of seed grants were deemed most important and feasible. We can contrast these findings with the results specific to our private university, whose participants rated items pertaining to collaboration and networking as most important and feasible. Participants from the hospital systems also identified seed grants for new collaborations as most important and feasible and for this group mentoring of junior faculty also appeared at the top. Faculty participants from academic institutions rated reduction in administrative barriers to collaboration higher in importance, while administrators and staff viewed the promotion of technical and analytic resources as a most important idea. It was observed that those without prior research funding rated the need to improve pre-award support for grant applications as a most important item. All of these contrasts were presented for consideration by stakeholders.

A final means for organizing the findings with an aim to promote action is presented in Table 3, which lists the ten ideas that participants rated as most important to enhancing CTR in Rhode Island, regardless of feasibility ratings. The most important items were providing seed grants, creating a common IRB, and developing blanket collaboration agreements between institutions in the Rhode Island CTR network.

**Table 3.** Top 10 ideas rated as most important towards increasing the quality and quantity of clinical and translational research

Rank	How can we increase the quality and quantity of CTR in RI?
1	Provide seed grants to encourage new collaborations across basic science and clinical faculty
2	Have a common Institutional Review Board (IRB) and Institutional Animal Care and Use Committee (IACUC) between Brown, Lifespan hospitals, and Care New England (CNE) hospitals
3	Create blanket collaboration agreements between institutions within the RI CTR network
4	Reduce administrative barriers to collaboration
5	Improve support for grant writing
6	Improve pre-award support for grant applications
7	Create recurring networking opportunities that connect researchers from different domains
8	Create a unified statewide directory of researcher expertise and projects
9	Connect researchers with common interests
10	Ensure that junior faculty have experienced mentors with a track record of funding

*Note:* CTR, clinical translational research; RI, Rhode Island.

## Discussion

The purpose of Group Concept Mapping is to represent the diverse perspectives of multiple stakeholders through a participant-authored, engaging approach (Trochim, 1989). For an evaluation with a participatory orientation, this method promised to go beyond providing information by stimulating discussion and informing action. Our results are specific to and discussed in the context of RI; however, it is important to note that the GCM process and the actions steps we took following the study are all likely achievable in a range of national, state, local, and institutional environments. As translational research continues to strive to understand assets and strengths and to address health needs across a diverse spectrum of stakeholders, communities and populations, engaging highly accessible participatory methods such as GCM will be critical.

The self-selection that followed the broad dissemination of our focus prompt is undeniable, but also speaks to the very nature of the task to increase investment and engagement of our stakeholders. The GCM activity included representatives from all of the participating organizations who comprised the demographic characteristics we thought most relevant to our purpose. The sorting exercise helped us to understand the functional and structural similarities of the generated ideas, and it provided a collective viewpoint of participants. Once clusters had been identified, ratings of importance and feasibility facilitated a better-informed discussion among stakeholders of means to accomplish the aims of our grant and potential action steps to follow. All of the 78 ideas were rated at least somewhat important (*M* > 2.85 out of 5.00) by our cross-section of stakeholders. Items that were rated most important were generally affirming of our project’s programmatic thrusts (e.g., more seed funding, ways to facilitate collaboration among individual researchers, better mentoring opportunities), and added a priority for breaking down barriers to inter-institutional collaboration and IRB agreements. Ideas supporting access to large datasets, statistical software, and support for research design and analyses were also perceived to be important, and those too are linked to major components of the project.

The particular circumstances of Rhode Island must be considered in the interpretation of results. These include a geographically compact base in which the participating institutions are independent. The two universities answer to different authorities, and the three health care organizations sometimes compete, and are only loosely affiliated with the universities. These circumstances likely contributed to the high degree of agreement that institutional collaboration is the most desired component of translational science enhancements in the state of Rhode Island. Though key differences in relative rankings were identified, there was agreement across institutions, researcher funding experience, and academic rank or role, that funding, networking and building collaborative connections across researchers and institutions were the most important ways to improve clinical and translational research in our state. However, differences were observed in terms of the relative ranking of the importance of community engagement by researchers and administrators. The different priority assigned to community engagement by the researchers and administrators is interesting, and we can only speculate on the viewpoints that might have contributed to this difference. The administrators may have understood that access to larger patient populations and enhanced connections to community groups should, in the longer term, strengthen institutional research capacity, while the researchers may have felt that they already had access to the patient populations they needed. Administrators may also be more attuned to NIH priorities for more effective connection of resources to the needs and concerns of diverse communities and the lack of such connection based on their reporting to NIH. The administrators’ perspectives seem prescient, given the emphasis that NIGMS has placed on community engagement in their current iteration of the CTR program. This finding may well extend to other CTR-supporting settings, and we encourage evaluators in other programs to examine the generalizability of this contrast in priorities as well as our more general picture of means for enhancing CTR.

The participatory nature of GCM lends itself to a high utility of results, and the evaluation team has also taken a series of steps to promote acting upon what has been learned. Graphic presentations made possible by the software (i.e., pattern match diagrams and go zone maps) have been featured, and we have also added a “top 10” chart ranking ideas by degree of importance alone and regardless of feasibility. After presenting these results to our operations committee, which represents the internal leadership across program components we then discussed the findings with our internal advisory committee, which brings together leadership from across the state, calling for suggestions of the best ways to move forward, particularly on those top ten items. The internal advisory committee consists of influential representatives of the institutions involved with the program (e.g., college deans, research directors) who typically have more power to implement change. They generated a number of suggestions for action, both orally and in writing, recognizing the need for work within and between their home institutions to foster collaboration. Following those meetings, we disseminated the results to GCM participants and others widely, in an electronic newsletter and in webinar presentation promoted through the Advance-CTR website (webinar available here: https://www.youtube.com/watch?v=nJ9yAj-u9NY).

Those who viewed that presentation (*n* = 311, December, 2020) were also asked to participate in a follow-up survey calling for their own suggestions and feedback based on the results presented in the webinar. We have also incorporated the GCM-generated ideas into our biannual reporting to project component leadership, identifying particular ideas contributed by GCM participants that seemed most relevant for each component activity. We then assisted these leaders in addressing these statements in their planning and programming. In many cases, the ideas validated activities that had already been prioritized, while other ideas added new possibilities for extending component missions and reach, and for refining current programming.

Several examples are provided here to highlight the direction of changes and initiatives. The top ranked idea, *to provide seed grants to encourage new collaborations across basic science and clinical faculty*, was already embedded in the project with several types of pilot award programs that prioritized and often mandated cross-institution investigators, as were “team science” workshops to facilitate interdisciplinary collaborations. However, building and maintaining those connections across institutions remains a challenge. Institutional leaders represented on the internal advisory committee have recognized that they play a key role in facilitating interdisciplinary and inter-institution collaboration and networking. State leadership reported a high degree of confidence that they could make progress on this front and the evaluation team will plan how to record and measure such efforts as they are implemented.

The desire to have a common IRB between institutions was also rated as highly important. Participants perceived this item to have low feasibility, indicating that there is recognition among researchers and administrators that this is a complex issue, despite widespread agreement that a common IRB would expedite and enhance clinical translation of research. Challenges regarding collaboration agreements across the project’s member organizations, as well as administrative barriers, were also considered important to address. We engaged our internal advisory committee to help us identify the most problematic administrative aspects to inter-institutional collaboration. The need for enhanced pre-award support, including grant writing, caused us to ask how Advance-CTR services and educational offerings can best be integrated with existing institutional resources for research supports and development. To promote networking opportunities, we are creating a unified statewide directory of researcher expertise and projects and have also started to track inter-institutional collaboration.

As illustrated by the contrasts between administrators and researchers in the pattern-matching rankings (Fig. 2), both the overlap and the differences in priorities aid in our understanding of which priorities should be considered for action. Administrators, for example, might recognize a need to clarify the importance of community engagement, and devise more accessible mechanisms for helping researchers to achieve this, while researchers may begin to perceive community engagement as more important if they receive explicit communication that federal funding agencies are prioritizing and even mandating these efforts. Effective mentoring was also rated as highly important to increase the quality and quantity of CTR in RI, particularly in the provision of experienced mentors with a track record of funding. Contrasting these ratings across institutions helped to highlight where mentoring needs were most acute. This led to several follow-up questions: what are the expectations of institutions, principal investigators (PIs), and junior investigators regarding mentorship? How can Advance-CTR help to facilitate better mentoring relationships that help lead to a more sustainable research infrastructure in the state? How feasible are opportunities for cross-institution mentoring? Addressing these questions aided our evaluation team in devising key indicators of successful mentorship, which is an important aspect of the CTR infrastructure.

## Conclusions and Lessons Learned

Our use of the GCM method benefited from the geographic proximity of the participants. Travel to the retreat to participate in the sorting and rating tasks in person was relatively easy, and personal relationships were leveraged to yield ample participation in the idea generation task. Key decisions made in our approach involved devising a suitable focus prompt, determining the duration of the idea generation period, and developing an itinerary for the retreat that included additional participatory activities to complement the individualized sorting and rating tasks, for which we provided 90 minutes’ time. In retrospect, providing additional weeks for idea generation might have yielded a greater number of unique ideas, and an augmented effort towards recruitment for the retreat might have increased the level of participation and organizational representation. Furthermore, now knowing that the ideas generated were all rated by our community to be at least somewhat important, we may have opted to conceptualize a more specific rating scale to better discriminate among degrees of “importance.” Yet overall, we considered the GCM activity to be highly productive, yielding important insights that continue to contribute substantially to the direction of the Advance-CTR program. It is not uncommon to be in meetings with project leadership who cite the GCM results as support for or against priorities, ideas, programs, etc., and that is extremely powerful!

Applying GCM to our state’s CTR network has enabled our project leadership to better understand stakeholder-perceived priorities, and to act on ideas and aims most relevant to researchers in the state. The affiliated organizations have been stimulated to consider their own contributions to the problems and the solutions, recognizing the challenges of policy change and budgetary stumbling blocks. Aspects of our program’s logic model have been reinforced and in some cases elaborated (e.g., the outcomes associated with inter-institutional collaboration). We found the GCM methodology to be highly participatory and effective at engaging our research community, and gained valuable insights that affirmed current services, programs, and initiatives; generated new ideas; and highlighted research barriers and needs. Furthermore, we have been able to address challenges unique to specific institutions in the state.

We have presented these results to project leadership, deans and research administrators at our state’s academic institutions, leadership within the state’s hospital systems, the research community in Rhode Island, the national network of CTR and CTSA evaluators, program officers at NIH, and a national conference of evaluators. It is important to note that while some of these stakeholder groups generally have very good understanding of research methods and statistical processes, others have very little or no training in these areas. The nature of the results presented and the ease of the visuals generated foster understanding and comprehension among very diverse audiences. It is hard to act on results one cannot access or understand and GCM reduces that research and statistics-based language barrier to action. Centered on our own program, we are pleased with the degree of enthusiasm expressed for the use of this method and continually seek feedback for ways we may be able to address ideas generated and deemed important by our Rhode Island health research community. We strongly encourage evaluators from other translational research programs to apply the GCM approach to determine how their program can better address the needs of their constituents. Broader application of this approach will also help to determine if our specific findings are generalizable or unique and will provide important data to inform national leadership to promote greater successes in research that improve population health.
